# Efficient Carrier Injection, Transport, Relaxation, and Recombination Associated with a Stronger Carrier Localization and a Low Polarization Effect of Nonpolar *m*-plane InGaN/GaN Light-Emitting Diodes

**DOI:** 10.1186/s11671-017-2087-8

**Published:** 2017-04-27

**Authors:** Fann-Wei Yang, Yu-Siang You, Shih-Wei Feng

**Affiliations:** 1 Department of Electronic Engineering, Southern Taiwan University of Science and Technology, Tainan, Taiwan Republic of China; 2 Department of Applied Physics, National University of Kaohsiung, No.700, Kaohsiung University Rd., Nanzih District, 811, Kaohsiung, Taiwan Republic of China

**Keywords:** Time-resolved electroluminescence (TR﻿EL), Carrier transport, Polarization effect, Nonpolar *m*-plane InGaN/GaN LED

## Abstract

Based on time-resolved electroluminescence (TREL) measurement, more efficient carrier injection, transport, relaxation, and recombination associated with a stronger carrier localization and a low polarization effect in a nonpolar *m*-plane InGaN/GaN light emitting diode (*m*-LED), compared with those in a polar *c*-LED, are reported. With a higher applied voltage in the *c*-LED, decreasing response time and rising time improve device performance, but a longer recombination time degrades luminescence efficiency. By using an *m*-LED with a stronger carrier localization and a low polarization effect, shorter response, rising, and recombination times provide more efficient carrier injection, transport, relaxation, and recombination. These advantages can be realized for high-power and high-speed flash LEDs. In addition, with a weaker carrier localization and a polarization effect in the *c*-LED, the slower radiative and faster nonradiative decay rates at a larger applied voltage result in the slower total decay rate and the lower luminescence efficiency. For the *m*-LED at a higher applied voltage, a slow decreasing nonradiative decay rate is beneficial to device performance, while the more slowly decreasing and overall faster radiative decay rate of the *m*-LED than that of the *c*-LED demonstrates that a stronger carrier localization and a reduced polarization effect are efficient for carrier recombination. The resulting recombination dynamics are correlated with the device characteristics and performance of the *c*- and *m*-LEDs.

## Background

Nitride-semiconductor light-emitting diodes (LEDs) have been commercialized for solid state lighting [1, 2]. The polar *c*-plane LED (*c*-LED) suffers from a well-known efficiency droop, i.e., quantum efficiency reaching maximum at a very low current density and then gradual reduction under a higher injected current [3–13]. Polarization effects induced quantum-confined Stark effect (QCSE) [3–8], electron leakage [9, 10], poor hole injection efficiency [11–13], and nonuniform hole distribution among the quantum wells have been suggested to explain the origin of efficiency droop. Auger recombination [14, 15] and carrier delocalization from indium-rich regions to dislocations [16] have also been proposed. The growth of III-nitrides along the nonpolar *m*- and *a*-axes [11, 17, 18], semipolar axis [19], and -*c*-axis (*nitrogen*-polar) [20] can overcome polarization effects. Without polarization effects in a nonpolar *m*-plane LED (*m*-LED), more uniform hole distribution among the wells and suppressed electron overflow out of the electron blocking layer can significantly reduce the electron leakage current and efficiency droop [11]. Without a QCSE, the flat energy band condition increases the overlap integral of the electron and hole wavefunctions, leading a higher recombination efficiency in nonpolar InGaN LED [11, 17, 18].

Time-resolved electroluminescence (TREL) can explore the dynamic EL behaviors of carrier injection, carrier transport, carrier relaxation into active region, and carrier recombination at the excited states in LEDs [20]. By TREL measurement, *nitrogen*-polar InGaN/GaN LEDs with the opposite polarity were shown to provide the advantages of more efficient carrier relaxation and carrier recombination [20]. However, carrier transport and recombination dynamics associated with a lower polarization effect in the *m*-LED are not well explored.

This study reports carrier transport and recombination dynamics associated with a stronger carrier localization and a low-polarization effect of the *m*-LED in comparison with those of the *c*-LED by using currentz-voltage (*I-V*), electroluminescence (EL), external quantum efficiency (EQE), and TREL measurements. By using an *m*-LED to overcome the polarization effect, shorter response, rising, and recombination times demonstrate that a stronger carrier localization and a reduced polarization effect are efficient for carrier injection, transport, relaxation, and recombination. In addition, due to a weaker carrier localization and a polarization effect in the *c*-LED, the slower radiative and faster nonradiative decay rates at a larger applied voltage result in the slower total decay rate and the lower recombination efficiency. At a larger applied voltage, the more slowly decreasing and overall faster radiative decay rate of the *m*-LED than that of the *c*-LED demonstrates that a stronger carrier localization and a reduced polarization effect are efficient for carrier recombination.

This paper is organized as follows. In section 2, methods are described. In section 3, results and discussions are reported. Finally, conclusions will be drawn in section 4.

## Methods

The *c*- and *m*-LEDs were simultaneously grown on the *c*- and *m*-plane sapphire substrates, respectively, by a metal organic chemical vapor deposition (MOCVD) reactor. The LED structure comprises a 25-nm low-temperature (LT) GaN buffer layer, a 3.00-μm undoped GaN layer, a 1.50-μm *n*-type Si-doped GaN contact layer, 10-pair InGaN/GaN multiple quantum wells (MQWs) (3 nm QW, 10 nm barrier), an 15 nm AlGaN electron blocking layer, and a 0.25-μm Mg-doped *p*-GaN contact layer. The 25-nm LT-GaN buffers were grown on nitridized sapphires at 600 °C in H_2_. Except for the substrates, the structures of the two LEDs are the same.

*I-V*, EL spectrum, and EQE were measured with a source meter (Keithley 2614B), spectrometer (Ocean Optics, resolution 0.3 nm), calibrated integrating sphere, and power meter. Without an ohmic contact, an indium dot melting on the sample surface was used as a contact for the probe station. For TREL measurement, a pulse generator (Tektronix AFG3152C) was used to generate 2.0–5.0 V, 0.5 μs pulse width, and 1 kHz repetition rate voltage pulses to the LEDs. The light output was focused and detected by a photosensor module containing a metal package PMT and a high-voltage power supply circuit (Hamamatsu H10721-210) operating directly on the surface of each LED. The same PMT voltages of the TREL measurement were applied for the two LEDs. The parasitic capacitance in the test circuit would introduce the same RC delay for the two LEDs. The transit EL signals were recorded by a digital oscilloscope (Agilent DSO 6052A) with a 500 MHz bandwidth. The overall resolution of TREL system is less than 2 ns. The detailed measurement was described in our previous study [20].

## Results and Discussions

### EL Spectra and EL Peak Position

**Fig. 1:**
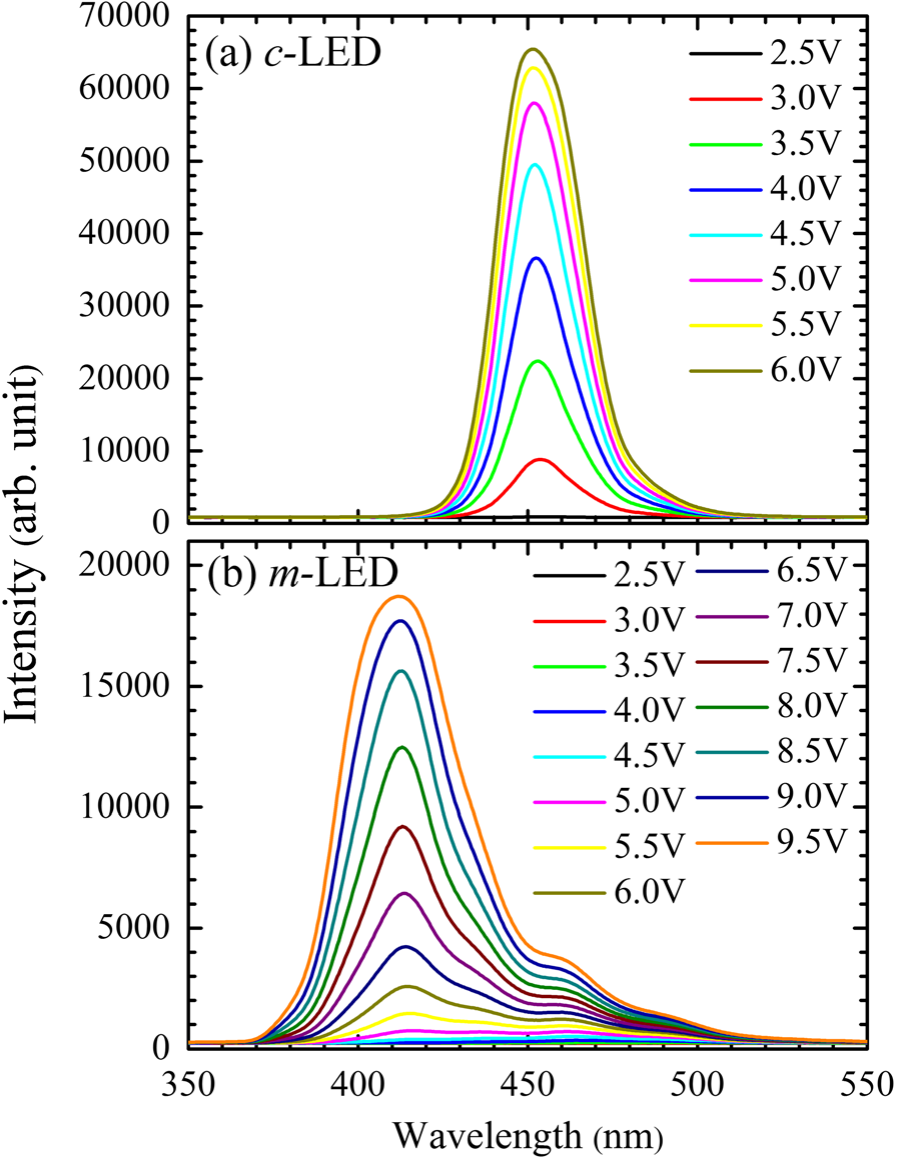
EL spectra for the (**a**) *c*- and (**b**) *m*-LEDs

Figure 1a, b shows the EL spectra for the *c*- and *m*-LEDs, respectively. EL emission wavelength for the *m*-LED is much shorter than that for the *c*-LED. EL spectra of the *c*-LED just coincide with the lower energy shoulder of that for the *m*-LED. Both carrier localization effect [21–23] and QCSE [3–8] in the *c*-LED contribute to luminescence. The large difference of EL peak positions for the *m*-LED from that for the *c*-LED can’t be discussed by only one effect. It can be due to the weaker QCSE or lower indium contents in the MQWs for the *m*-LED. The larger bandwidth of EL and the lower energy shoulder in the *m*-LED represent a stronger indium composition fluctuation and a stronger carrier localization. The broader bandwidth of EL in the *m*-LED could be due to the luminescence contributions from different indium compositions and indium clusters.

**Fig. 2:**
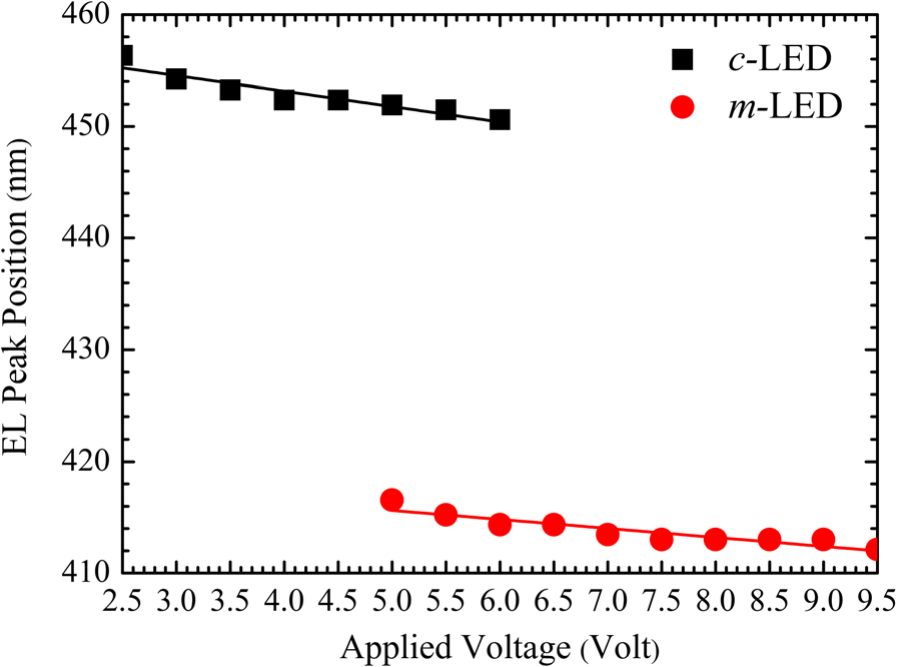
EL peak position as a function of CW applied voltage for the *c*- and *m*-LEDs. EL peak positions are linearly fitted for comparisons

In addition, Fig. 2 shows EL peak position as a function of applied voltage for the two LEDs. EL peak positions are linearly fitted for comparisons. The slightly blue-shifted EL peak position at a higher applied voltage for the *m*-LED could be due to the band-filling effect of higher energy states and a reduced polarization effect upon a forward bias.

### *I*-*V* and (EQE)

**Fig. 3:**
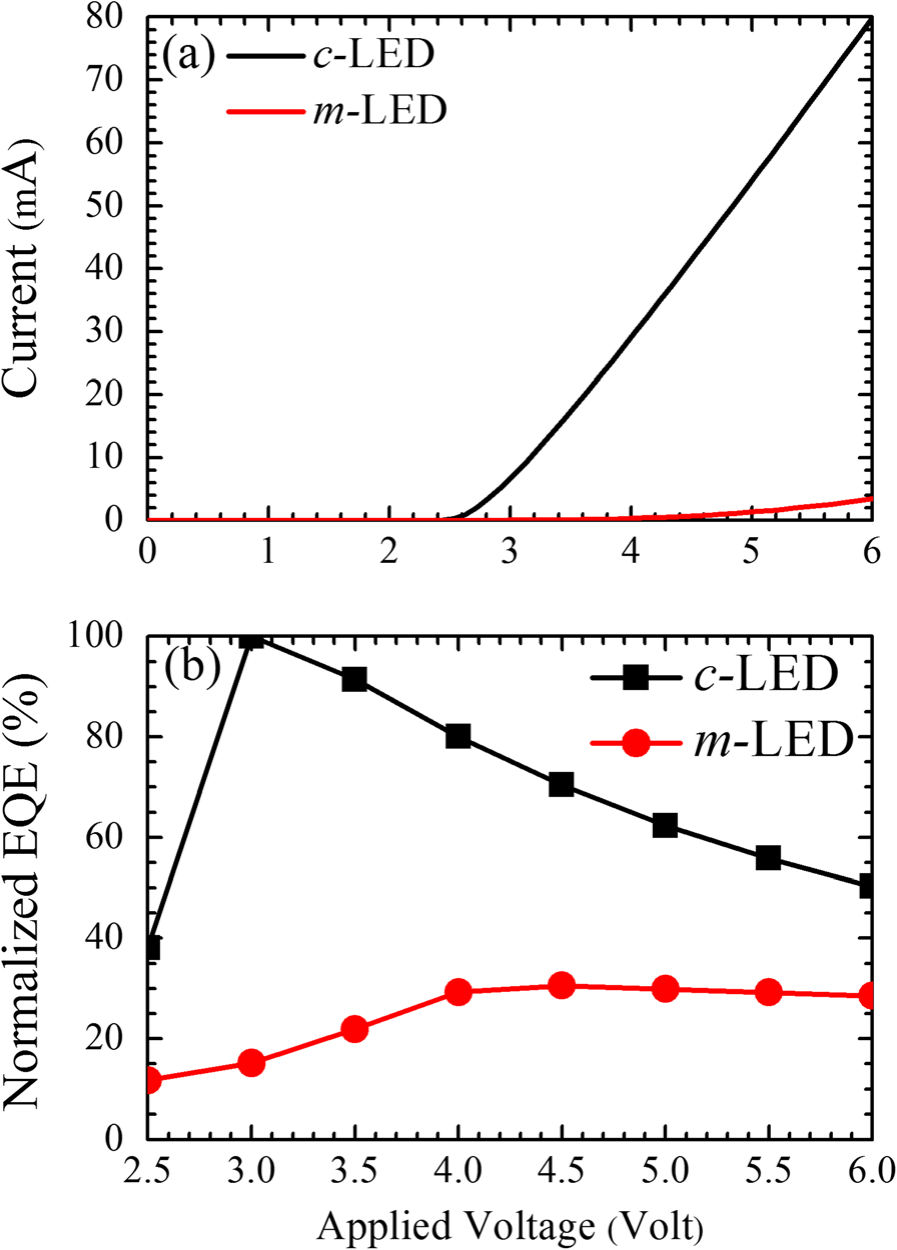
**a** Current (*I*) and (**b**) normalized EQE as functions of applied voltage (*V*) for the *c*- and *m*-LEDs

Figure 3a, b shows the *I*-*V* characteristics and normalized EQE, respectively, of the two LEDs. The turn-on voltages of *c*- and *m*-LEDs are 2.5 and 3.5 V, respectively. Although a slightly larger threshold, smaller slope of current under forward bias, and lower EQE of the *m*-LED suggest poorer device quality, a stronger carrier localization and a reduced polarization effect help the *m*-LED to exhibit better carrier transport and recombination dynamics, as shown later.

### TREL and Decay Rates

**Fig. 4:**
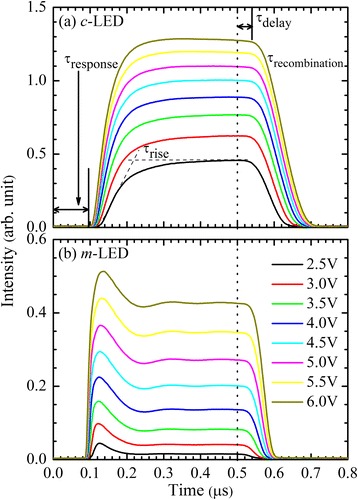
TREL transit profiles for the (**a**) *c*- and (**b**) *m*-plane LEDs, with the *vertical dotted line* indicating the pulse voltage switching off. Response (*τ*_response_), rise (*τ*_rise_), delay (*τ*_delay_), and recombination (*τ*_*recombination*_) times are shown in **a**

Figure 4a, b shows the TREL transit profiles of the *c*- and *m*-LEDs, respectively. After the voltage pulses are applied, different rising transit profiles for the two LEDs imply different behaviors of carrier injection and carrier transport. The rising part of the transit EL intensity of the *c*-LED shows a slower response and rise. Similar temporal behavior was observed in transition from free carrier to deep acceptor levels of a GaN/AlGaN LED [24] and in the transit EL for the long-wavelength shoulder of phonon-assisted EL in Si [25]. Because the EL comes from the carrier recombination at lower energy states, the slow rise can be due to the carrier capture process from higher energy states into lower energy states. In contrast to the *c*-LED, the rising part of transit EL intensity for the *m*-LED shows a shorter response, steeply rising, and then decay within the first 100 ns. Similar temporal behavior, attributed to band-to-band emission, was observed in EL peak at 285 nm of a GaN/AlGaN LED [24]. Without QCSE in the *m*-LED, carriers may transport from high energy states to lower energy states. It is noted that PMT has a better sensitivity and can amplify low signals. The intensity ratios of TREL transit profiles under the same bias for the two LEDs are different from those of EL spectra.

As shown in Fig. 4, the response time (*τ*_*response*_) can be determined by the time delay between addressing the device with a short voltage pulse and the appearance of EL. The rising time (*τ*_rise_) is defined by the intercept of the tangents. Delay time (*τ*_delay_) is the time delay in reaching both the maximum intensity of transit EL and the subsequent decay, when the voltage pulse is switched off (indicated with the vertical dotted lines). The recombination time (*τ*_*recombination*_) can be determined by fitting the decay profile with a single exponential.

**Fig. 5:**
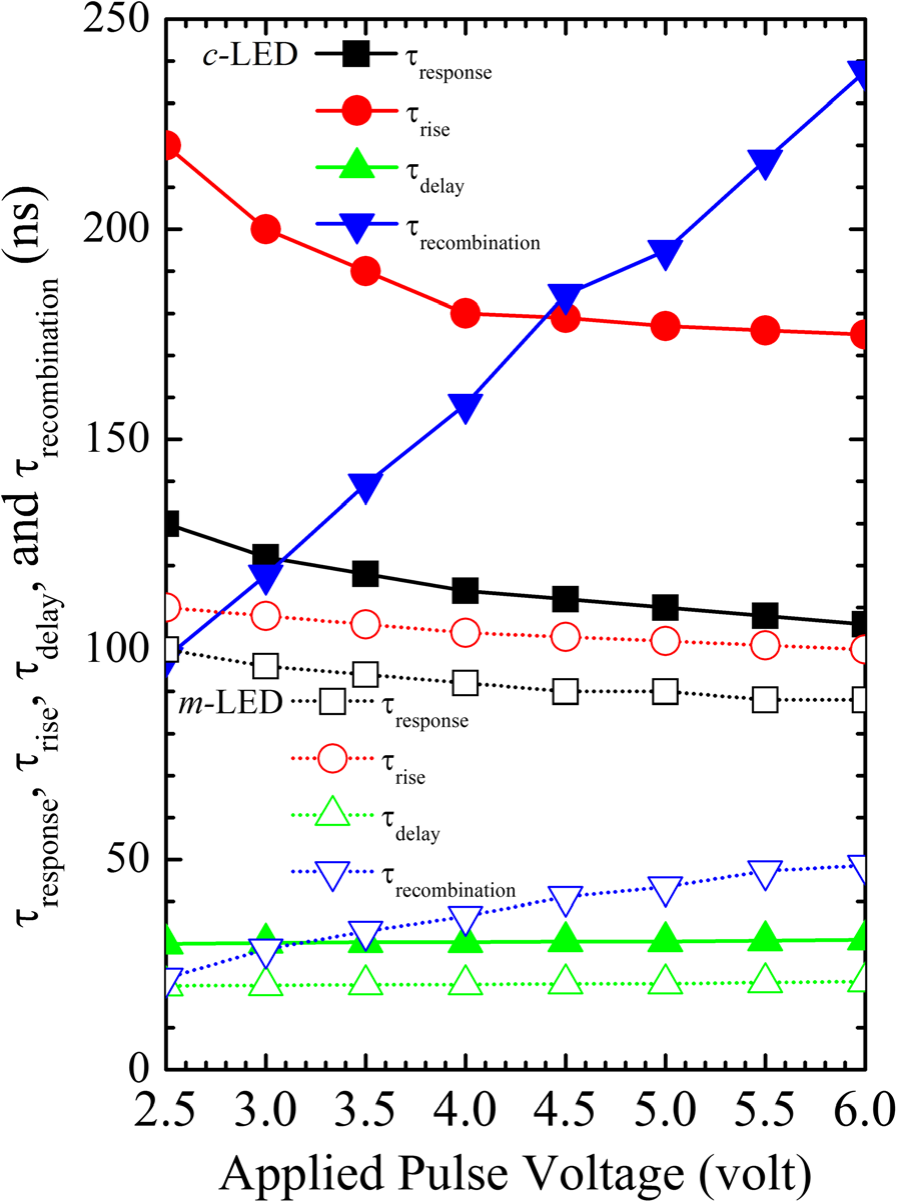
Response (*τ*_response_), rise (*τ*_rise_), delay (*τ*_delay_), and recombination (*τ*_*recombination*_) times as functions of applied pulse voltage for the two LEDs

As shown in Fig. 5, *τ*_*response*_ can be described as [20]:

1 $$ {\tau}_{response}={\tau}_{injection}+{\tau}_{transport}+{\tau}_{relaxation}+\tau {\textstyle,}_{recombination}, $$

where *τ*_*injection*_, *τ*_*transport*_, *τ*_*relaxation*_, and *τ* ' _*recombinaiton*_ are the carrier injection time to the ohmic contact, carrier transport time from the ohmic contact to the active region, carrier relaxation time from the active region to the MQWs, and carrier recombination time in energy states, respectively. With the same contact material for the two samples, *τ*_*injection*_ can be assumed to be the same. Also, due to the faster mobility of nonpolar *m*-plane GaN [26], *τ*_*transport*_ of the *m*-LED should be shorter. Without polarization charges, a lower potential barrier makes the carrier relaxation efficiency of the *m*-LED better than that of the *c*-LED [27], so *τ*_*relaxation*_ of the *m*-LED should be shorter. The weaker polarization effects in the *m*-LED increases the overlap of electron and hole wavefunctions and the *τ* ' _*recombinaiton*_ is expected to be shorter, as shown later. This suggests that a weaker polarization effect in the *m*-LED helps carrier relaxation into MQWs and a stronger carrier localization helps carrier recombination in higher energy states. Hence, the shorter *τ*_*response*_ of the *m*-LED should be due to shorter *τ*_*transport*_, *τ*_*relaxation*_, and *τ* ' _*recombinaiton*_.

At the same time, at a higher applied voltage in the *m*-LED, shorter *τ*_*response*_, *τ*_rise_, *τ*_delay_, and *τ*_*recombination*_ of the *m*-LED than those of the *c*-LED demonstrate that a stronger carrier localization and a reduced polarization effect of the *m*-LED are more efficient for carrier injection, transport, relaxation, and recombination. The advantages can be realized for applications of high-power and high-speed flash LEDs. Although a longer *τ*_*recombination*_ at a higher applied voltage shows a slower carrier recombination rate, it is shorter than *τ*_*response*_ and *τ*_rise_.

In a previous study, bias-dependent EL, EQE, and TREL measurements were conducted to compare the effects of carrier localization and QCSE on carrier transport and recombination dynamics of *Ga*- and *N*-polar LEDs [20]. It is noted that the external bias can change the potential energy band profile, so it can change the overlap integral of the electron and hole wavefunctions. With the same external bias, the polarization effect in the *c*-LED and the reduced polarization effect in the *m*-LED could be compared. The polarization-associated recombination dynamics in the *c*- and *m*-plane LEDs were also revealed by using bias-dependent EL, EQE, and TREL measurements.

In addition, the injected current density may affect the carrier transport and recombination dynamics in LEDs. Because the carrier recombination is mainly from the ground states in the quantum wells, the injected current density may not affect it much. Under the same current density of the *c*-LED at 3.0V and the *m*-LED at 6.0V, shorter *τ*_*response*_, *τ*_rise_, *τ*_delay_, and *τ*_*recombination*_ of the *m*-LED than those of the *c*-LED can be confirmed to demonstrate that a stronger carrier localization and a reduced polarization effect are efficient for carrier injection, transport, relaxation, and recombination.

The decay time (*τ*) is the reciprocal of the total decay rate (*κ* = 1/*τ*), which is the sum of the radiative and nonradiative decay rates [1]:

2 $$ \kappa ={\kappa}_r+{\kappa}_{nr} = \frac{1}{\tau}, $$

where *κ*, *κ*_r_, and *κ*_nr_ are the total, radiative, and nonradiative decay rates, respectively. In addition, due to the same device structures, the extraction efficiencies of the two LEDs are assumed to be the same and the normalized EQE, as shown in Fig. 3b, can be regarded as the internal quantum efficiency, *η* [28]. *η* is described by the radiative decay rate over the total decay rate of recombination [1]:

3 $$ \eta =\frac{\kappa_r}{\kappa_r+{\kappa}_{nr}}=\frac{\kappa_r}{\kappa}. $$

*κ*_r_ and *κ*_nr_ can be calculated by solving equations (2) and (3).

**Fig. 6:**
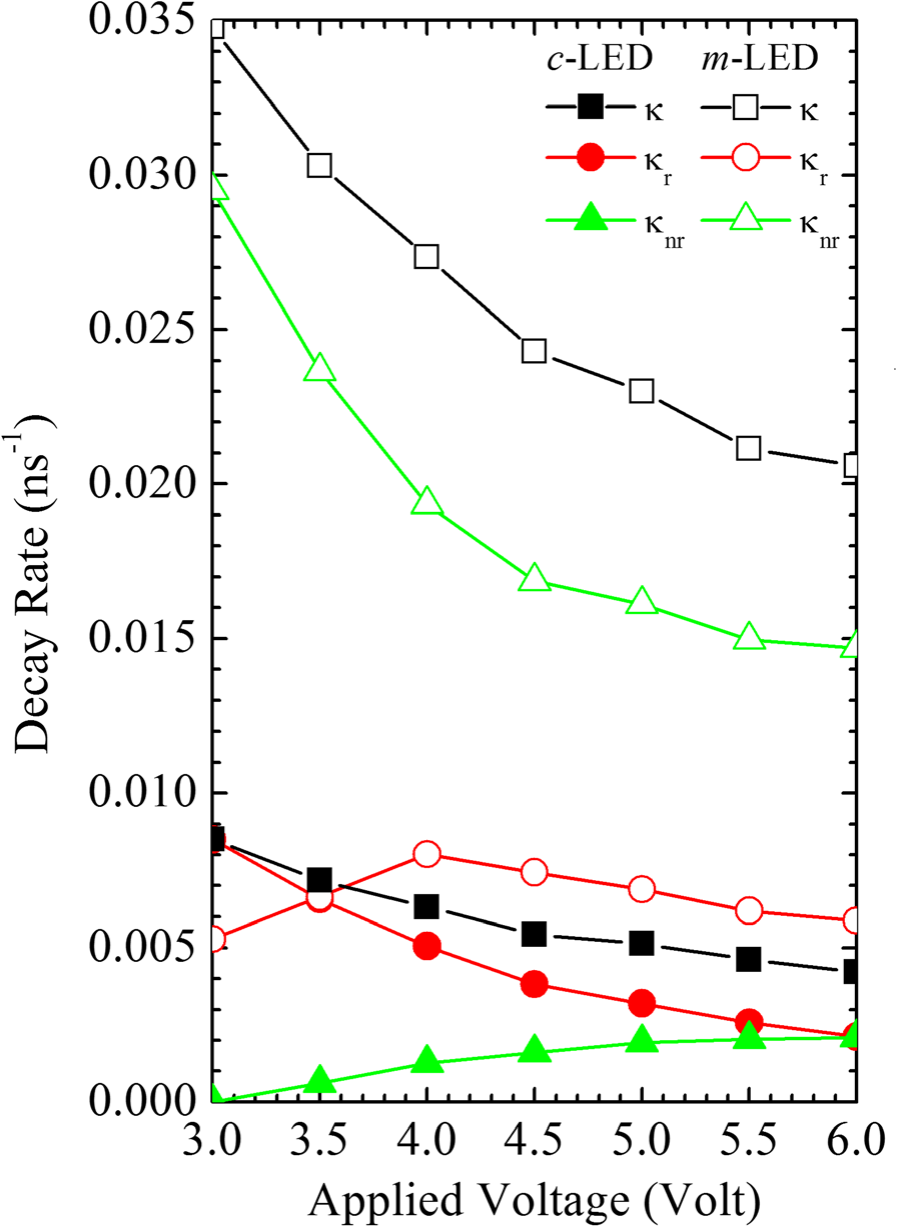
The total (*κ*), radiative (*κ*_r_), and nonradiative (*κ*_nr_) decay rates as functions of applied voltage for the *c*- (*filled symbols*) and *m*-LEDs (*unfilled symbols*)

*κ*, *κ*_r_, and *κ*_nr_ associated with recombination dynamics are shown in Fig. 6. For the *c*-LED at a lower applied voltage, a smaller deviation between *κ* and *κ*_r_ shows that the recombination dynamics are dominated by the *κ*_r_. With a higher applied voltage, the slower *κ*_r_ and faster *κ*_nr_ result in the slower *κ*.

For the *m*-LED at a higher applied voltage, a steeply decreasing *κ*_nr_ is beneficial to device performance. In spite of the lower *η* of the *m*-LED, the slower decreasing *κ*_r_ and overall faster *κ*_r_ of the *m*-LED than that of the *c*-LED demonstrates that a stronger carrier localization and a reduced polarization effect are efficient for carrier recombination. Because the *c*- and *m*-LEDs were simultaneously grown on the *c*- and *m*-plane sapphire substrates, respectively, the un-optimized growth condition for the *m*-LED leads to a faster *κ*_nr_ than *κ*_r_. The *κ*_r_ should be enhanced by optimizing growth conditions for the *m*-LED separately. Also, an *m*-LED can be fabricated on an *m*-plane GaN substrate [29]. In addition, under the same current density of the *c*-LED at 3.0V and the *m*-LED at 6.0V, the faster *κ*_r_ of the *m*-LED than that of the *c*-LED can be confirmed to demonstrate that a stronger carrier localization and a reduced polarization effect are efficient for carrier recombination, while the faster *κ*_nr_ of the *m*-LED can be due to an un-optimized growth condition and a higher defect density. The resulting recombination dynamics are correlated with the device characteristics and performance of the *c*- and *m*-LEDs.

## Conclusions

In summary, this study reveals that by using an *m*-LED with a stronger carrier localization and a weaker polarization effect, shorter response, rising, and recombination times provide more efficient carrier injection, transport, relaxation, and recombination. By optimizing growth conditions to enhance the radiative recombination, the advantages in the *m*-LED can be realized for the applications of high-power and high-speed flash LEDs. In addition, due to a weaker carrier localization and a larger polarization effect in the *c*-LED, the slower radiative and faster nonradiative decay rates at a higher applied voltage together result in the slower total decay rate and the lower recombination efficiency. With a higher applied voltage in the *m*-LED, a slow nonradiative decay rate is beneficial to device performance, while the more slowly decreasing and overall faster radiative decay rate of the *m*-LED compared to that of the *c*-LED demonstrates that a stronger carrier localization and a reduced polarization effect are efficient for carrier recombination. The resulting recombination dynamics are correlated with the device characteristics and performance of the *c*- and *m*-LEDs.
